# Considerable Variation of Antibacterial Activity of Cu Nanoparticles Suspensions Depending on the Storage Time, Dispersive Medium, and Particle Sizes

**DOI:** 10.1155/2015/412530

**Published:** 2015-08-03

**Authors:** Olga V. Zakharova, Anna Yu. Godymchuk, Alexander A. Gusev, Svyatoslav I. Gulchenko, Inna A. Vasyukova, Denis V. Kuznetsov

**Affiliations:** ^1^Tambov State University named after G.R. Derzhavin, 33 Internatsionalnaya street, Tambov 392000, Russia; ^2^National University of Science and Technology “MISIS”, 4 Leninsky pr., Moscow 119991, Russia; ^3^National Research Tomsk Polytechnic University, 30 Lenina Avenue, Tomsk 634050, Russia

## Abstract

Suspensions of Cu nanoparticles are promising for creating the new class of alternative antimicrobial products. In this study we examined copper nanoparticles of various sizes obtained by the method of wire electric explosion: nanopowder average size 50 nm (Cu 50) and 100 nm (Cu 100). The paper presents the complex study of the influence of physicochemical properties such as particle size and concentration of the freshly prepared and 24-hour suspensions of Cu nanoparticles in distilled water and physiological solution upon their toxicity to bacteria *E. coli* M-17. Ionic solution of Cu^2+^ and sodium dichloroisocyanurate was used for comparison study. It has been shown that decrease in the nanoparticle size leads to changes in the correlation between toxicity and concentration as toxicity peaks are observed at low concentrations (0.0001⋯0.01 mg/L). It has been observed that antibacterial properties of Cu 50 nanoparticle suspensions are ceased after 24-hour storage, while for Cu 100 suspensions no correlation between antibacterial properties and storage time has been noted. Cu 100 nanoparticle suspensions at 10 mg/L concentration display higher toxicity at substituting physiological solution for water than Cu 50 suspensions. Dependence of the toxicity on the mean particle aggregates size in suspension was not revealed.

## 1. Introduction

Nanotechnologies are expected to be developed at medicine, microelectronics, optics, catalysis, sensor analysis, and other manufacturing sectors [1–4]. Nowadays, creation of a new class of alternative antimicrobial agents may be one of the promising applications for metal nanoparticles [5–7] because of the increasing of antibiotic resistance in microorganisms [8, 9] presenting grave hazard for public healthcare [10].

At the present time a considerable number of papers studying antibacterial properties of metal nanoparticles [11, 12] pose a wide spectrum of antibacterial activity without resistance development in microbes [13–19]. Meanwhile, due to high biological activity, comparatively low cost, and ecological safety, copper nanoparticles can be considered as promising multifunctional antibacterial agents [20].

Several authors showed that nanosized cuprum particles display antimicrobial activity towards wide range of microorganisms, including pathogenic bacteria [21, 22]. It is important to note that antibacterial properties are displayed by cuprum nanoparticles [23–25], cuprum compounds [26–30], and complex nanomaterials containing cuprum [31–34].

Cytotoxicity of Cu nanoparticles results not only from the small size of the particles, high specific surface value, and close interaction with microbial membranes but also from formation of leached cuprum-peptide complexes leading to several-fold increase in reactive oxygen intermediate (ROI) generation, cell viability decrease, and general biomass growth suppression [35]. Storage of cuprum nanoparticle suspensions for some time may lead to Cu^2+^ ions release into the culture medium, as cuprum has high reaction activity [36].

It was also found that cuprum nanoparticles display on the one hand low toxicity towards humans and on the other hand show high antimicrobial effect towards the cells of test-cultures of gram-positive and gram-negative bacteria which makes them applicable for creating the new wound healing products [22]. By the example of* E. coli* it was shown that utilization of Cu nanoparticles is of high potential for creating new bactericidal dressings as human tissues are resistant to cuprum [37], while microorganisms are highly sensitive to it [38–40].

However, creation of such products is hampered by the lack of experimental data on the biological activity alteration when passing the upper nanoscale limit (100 nm) and at transferring from ion-molecule form to nanoscale through the lower limit (10 nm).

Studies of nanoparticle antimicrobial properties have made it possible to discover possible mechanisms of their toxic action including oxidative stress due to reactive oxygen intermediates generation, lipid peroxidation, protein oxidation and DNA degradation in cells, mechanical damage of cell membranes [41], and influence of some physicochemical characteristics such as size, shape, and production method upon the level of biocidal impact [5, 23, 42–45].

At the same time such factors as agglomeration and rapid oxidation have made this research area difficult. Although, some researches use stabilizers in order to eliminate these factors [46–48], joint use of nanoparticles and stabilizers may lead to synergistic effects. Besides, nanoparticle antibacterial properties are usually studied* in vitro* in water or agarous media but not in biological fluids. Moreover, dependence of nanoparticle suspensions toxicity upon their storage time is still unstudied. Thus, mechanisms which are connected with nanoparticles behavior in colloidal systems and essential for cytotoxicity are still undiscovered.

This paper shows alteration of copper nanoparticles bactericidal properties depending on physicochemical characteristics of the particles, dispersive medium, and suspension storage time.

## 2. Materials and Methods

### 2.1. Nanoparticles

**Figure 1:**
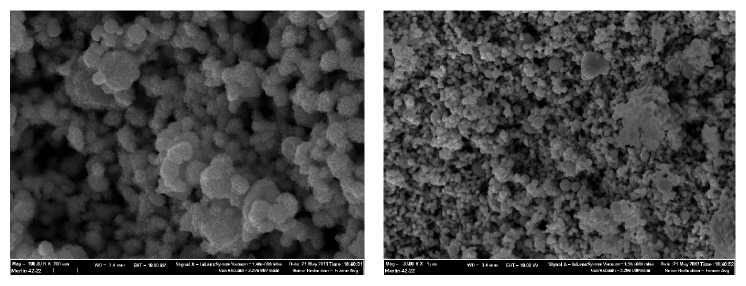
Electron micrograph of Cu 50 nanoparticles powders.

**Figure 2:**
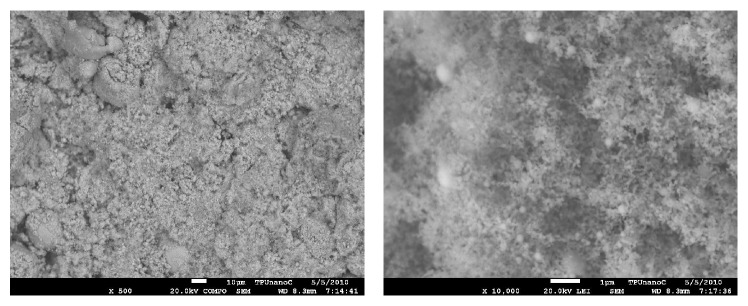
Electron micrograph of Cu 100 nanoparticles powders.

In this study we examined copper nanoparticles of various sizes obtained by the method of wire electric explosion in Ar-medium with additive of H_2_ (10 vol. %) at the pressure of 1.52·10^5^ Pa, capacitive storage charging voltage −24 kV (the wire diameter 0.3 mm, length 75 mm). To maintain metal stability to oxidation nanopowders were passivated by slow air oxidation (Advanced Powder Technologies LLC, Tomsk, Russian Federation [49]). According to the manufacturer arithmetic average size of the particles was 50 nm (Cu 50, specific surface area 12 m^2^/g) and 100 nm (Cu 100, specific surface area 6.8 m^2^/g); the nanoparticles were spherical in shape (Figures 1 and 2).

On the assumption that the shape of the particles is almost spherical, thickness of the oxidation film (*X*) on the particle surface was calculated using the following formulas:

(1) X=Rpart−RМe,Vpart=43·π·Rpart3,VМe=VpartυCu,RМe=34·VМeπ1/3,

where *R*_part_ is the particle radius, *V*_part_ is the particle volume, *V*_*М*e_ is the volume of the metallic sphere, *R*_*М*e_ is the radius of the metallic part, and *R*_part_ is the radius of the particle coated with oxidation film.

The calculation results for oxidation film for the initial samples are presented in Table 1.

The powders were stored in sealed glass vials at the temperature of 20 ± 1°С; the vials were opened not earlier than a week before the suspension preparation.

### 2.2. Nanoparticles Suspensions

The nanoparticle suspensions were prepared with doubly distilled water (рН = 7.1 ± 0.2,) and 9% NaCl solution (рН  7.1 ± 0.2). Precisely weighed quantities were determined using ViBRA HT analytical balance (Shinko Denshi, Japan, with precision ±0.0001 g), poured into extemporaneously prepared dispersion vehicle and stirred with a glass rod for 20 seconds. After stirring the suspensions were processed in Ultrasonic Cleaner CD-4800 (Codyson, China) for 40 seconds (70 W, 44 Hz, volume 1.4 L). Initial copper concentration in the solutions was 10 mg/L; the initial solutions were then diluted with doubly distilled water or physiological solution to prepare suspensions with copper concentrations of 1, 0.1, 0.01, 0.001, and 0.0001 mg/L.

The laboratory glassware for sample storage and biotesting was washed with mixture of potassium bichromate and sulphuric acid (chromic-sulphuric acid mixture). The glassware inner surfaces were gently wetted with chromic-sulphuric acid mixture and left for 2-3 hours; the glassware was then washed thoroughly with tap water, disacidified with sodium bicarbonate solution, and washed for 3-4 times with doubly distilled water.

Toxic properties were analyzed in freshly prepared (stored for no more than 1 hour) and stored for no more than 24-hour suspensions.

### 2.3. Comparison Solutions

#### 2.3.1. Cu^2+^ Ion Solutions

Solutions containing Cu^2+^ ions were used for comparison. Solutions were prepared by dissolving copper chloride (CuCl_2_ *∗*  2H_2_O, GOST 4167-74, Russian Federation) in doubly distilled water (рН = 7.1 ± 0.2) with conductivity of 0.2 *μ*S and in physiological solution (рН  7.1 ± 0.2).

#### 2.3.2. Sodium Dichloroisocyanurate Solutions

Aqueous solutions of sodium dichloroisocyanurate (C_3_Cl_2_N_3_NaO_3_, SDC) (NPF Praktika LLC, Russian Federation) were employed for positive control; they were also prepared with doubly distilled water and physiological solution. Sodium dichloroisocyanurate is widely used as a disinfectant efficient against gram-positive and gram-negative bacteria, viruses,* Candida* fungus, and dermatophytes [50].

Solutions of Cu^2+^ ions and sodium dichloroisocyanurate were prepared within concentration range of 0.0001⋯10 mg/L.

### 2.4. Toxicity Evaluation

Copper nanoparticle solutions toxicity was measured by bioluminescence technique used for microbiological and molecular genetic evaluation of nanomaterials influence on microbiocenosis species [51–54]. The method measures modifications in bioluminescence intensity of the genetically modified photobacteria strain* E. coli* M-17 influenced by nanoparticles present in the analyzed sample as compared with the control sample. Alteration in the bioluminescence intensity of the tested object in the analyzed sample as compared with the control sample containing no toxic agents was taken as effect criterion. Bioluminescence intensity reduces in proportion to toxic effect.

Toxic effect of the studied nanomaterial sample upon bacteria is determined by their bioluminescence inhibition after 30-minute exposure period. The quantitative test-reaction parameter assessment is expressed as a toxicity index *T* which is a nondimensional quantity determined from the formula *T* = 100(*I*_*o*_ − *I*)/*I*_*o*_, where *I*_*o*_ and *I* are luminous intensities of the control and tested samples accordingly, while the exposition time of the examined sample with the test-object is fixed.

The technique allows for three threshold levels of the toxicity index:

(1) acceptable degree when toxicity index *T* is in the range from 0 to 20;
(2) medium degree when toxicity index *T* is in the range from 20 to 50;
(3) high degree when toxicity index *T* equals or is higher than 50.

Negative toxicity index values are regarded as nontoxicity.

In the process of toxicity index evaluation parallel measurements of control and studied samples were carried out. For higher data reliability the number of repeat sample tests was increased up to 5 measurements.

The measurements were carried out using Biotox-10 specialized luminometer (Russian Federation). The first test phase was carried out straight after the solutions were prepared; the second phase was carried out in 24 hours. The solutions pH value was checked before each measurement using digital pH-meter рН-2005 SELECTA (Barcelona, Spain).

### 2.5. Measurement of the Dispersity

The particle/aggregate size distribution in the prepared suspensions was evaluated at the temperature of 25°С using the dynamic light scattering technique and the Malvern Zetasizer Nano device, USA (a helium-neon laser with the power of 4 mW and the 633 nm wave length). For these measurements, a versatile capillary U-shaped polystyrene cuvette was used. The dry cuvette was delicately washed with distilled water; then 1 mL of the suspension under study was poured in, avoiding air bubbles formation. Each measurement was repeated thrice. Based on the obtained size distribution; the average particle size at each point was calculated according to the following formula:

(2) $$\begin{array}{c} d_{av}=\sum d\frac{q\left(\%\right)}{100\left(\%\right)}, \end{array}$$

where *d* represents the particle size in dispersion and *q* is the differential percent of particles with size *d* in the dispersion.

## 3. Results and Discussion

Molecules interact with macroscopic bodies' surfaces according to the laws of molecular statistics, while in transition to nanoobjects and live cells statistics of comparatively large objects and their contact interactions acquire importance, in which case the particle roles change qualitatively: the larger ones may be considered as motionless while nanoparticles and metallic ions may be considered as more mobile than bacterial cells, as their dimensions (1–100 nm) are smaller than average* E. coli* cell size (1–3 *μ*m in length, 0.5–0.8 *μ*m in width). Owing to the fact that reactivity of the solids is in proportion to their surface area, other factors, including concentration being equal, the choice of copper configurations such as 50 nm and 100 nm copper suspensions and Cu^2+^ containing solutions, were justifiable for the study. Presumably high biological activity per unit mass was to be observed for all the chosen configurations as compared to larger particles.

### 3.1. Study of the Antibacterial Properties of Cu 50 Nanoparticle Suspensions

**Figure 3:**
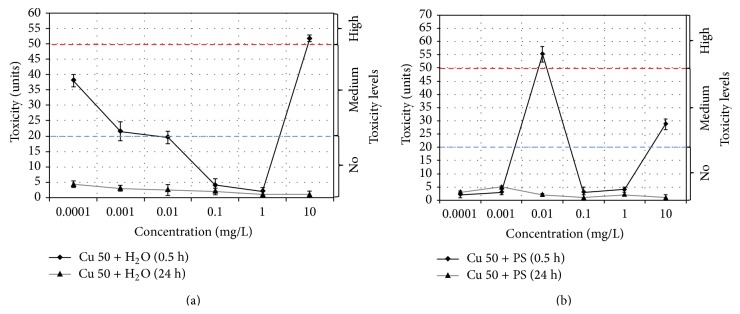
Changes in the toxicity index for Cu 50 nanoparticle suspensions based on (a) water, Cu 50 + H_2_O, and (b) physiological solution, Cu 50 + PS.

The study of the antibacterial properties of 0.5-hour aqueous Cu 50 nanoparticle suspensions has allowed us to discover that toxicity observed at the lowest concentrations of 0.0001 mg/L decreases from 40 to 20 units with nanoparticle concentration growth up to 0.01 mg/L, no toxicity is observed in suspensions with 0.1…1 mg/L concentration, and the maximum value (≈50 units) is recorded at 10 mg/L suspension (Figure 3(a)). The solutions pH value monitoring throughout the testing process indicated slight deviation of pH up to 7.6 from the normal values of 6.8–7.4 specifically in suspensions with 0.0001 and 0.001 mg/L concentrations (Table 2). The highest value of toxicity index in 10 mg/L suspension may be connected with increased concentration of Cu^2+^ ions dispersed in the solution as a result of the sample dissolution during its hour-long exposure [35, 55].

According to the experiment, 24-hour suspensions of Cu 50 particles display no antibacterial effect (Figure 3(a)) in the studied concentration interval. Lack of toxicity may be explained by the storage period as copper particles with size < 150 nm may oxidize to 11–14 wt.% developing on the surface oxide-hydroxide forms of copper blocking further dissolution [55].

Within the studied concentration range toxicity index for physiological solution (PS) medium displays the highest values in 0.5-hour suspensions at particle concentrations of 0.01 mg/L (>50 units) and 10 mg/L (≈30 units) (Figure 3(b)). 24-hour Cu 50 nanoparticle suspensions in physiological solution display no toxic effect on the tested object. Thus, the highest toxic effect is observed in freshly prepared Cu 50 nanoparticle suspensions. Minimum inhibitory concentrations (MIC) and Minimum bactericidal concentrations (MBC) were evaluated by means of the computational method [56]. Neither MIC nor MBC could be calculated for Cu 50 nanoparticles because of the nonlinear type of the dependence.

**Figure 4:**
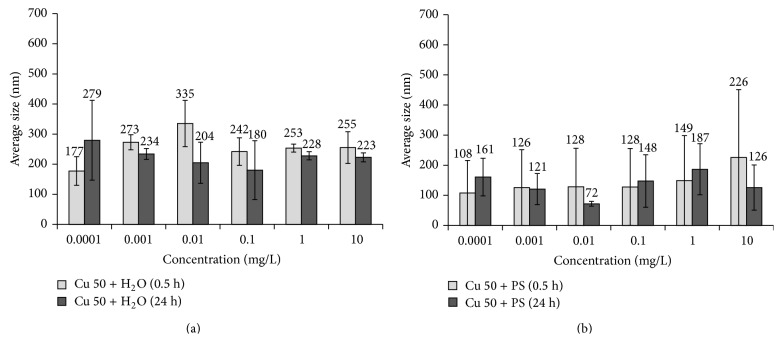
Changes in average size of Cu 50 nanoparticle suspensions based on (a) water, Cu 50 + H_2_O, and (b) physiological solution, Cu 50  +  PS.

Dispersion analysis of 0.5-hour aqueous Cu 50 nanoparticle suspensions showed insignificant alteration in particle aggregates sizes (*d*_av_); slight growth of *d*_av_ from 177 to 250–270 nm was observed with concentration increase. In 24 hours *d*_av_ of aggregates in suspensions decreases slightly; it may be connected with larger aggregates subsidence (Figure 4(a)). In physiological solution a tendency towards increase of *d*_av_ of the aggregates in 0.5-hour solutions from 108 to 226 nm was observed together with increase in concentration from 0.0001 to 10 mg/L, respectively (Figure 4(b)). In 24 hours *d*_av_ value changes insignificantly.

Comparison of toxicological and dispersion analysis data leads to suggestion that size of aggregates insignificantly changing with storage time and concentration growth is not the main reason for variations in antibacterial properties. It is probable that chemical state of the surface is much more important for toxicity level variations than dispersity.

### 3.2. Study of the Antibacterial Properties of Cu 100 Nanoparticle Suspensions

**Figure 5:**
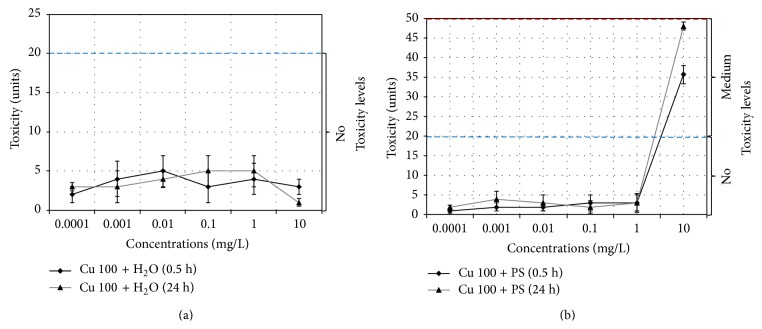
Changes in the toxicity index for Cu 100 nanoparticle suspensions based on (a) water, Cu 100 + H_2_O, and (b) physiological solution, Cu 100 + PS.

According to the data obtained from bioluminescence technique aqueous Cu 100 nanoparticle suspensions show no toxic effect on bacteria in the studied range of concentrations (Figure 5(a)). The same properties are characteristic for PS suspensions, excluding 10 mg/L suspensions where medium toxicity level of 35–50 units is observed (Figure 5(b)).

Minimum inhibitory concentration (MIC) was assessed for physiological solution: it is 4 mg/L for the freshly prepared suspension and 5 mg/L for the 24-hour suspension. Minimum bactericidal concentration (MBC) was not detected in the studied range of concentrations.

Data obtained from рН measurements display that slight shift in pH value up to 7.7 is observed only in 10 mg/L PS suspensions of Cu 100 nanoparticles (Table 3).

Comparative analysis of bactericidal properties of the particles in H_2_O and PS media shows increase of toxicity index in the presence of electrolyte. The received data corresponds with the previous experiments stating that presence of more polar solvent (Cl^−^ ions) increases the degree of Cu 100 particles dissolution [55]. Detachment of large amount of Cu^2+^ ions having higher diffusion activity than solid particles may lead to increase in cytotoxicity.

**Figure 6:**
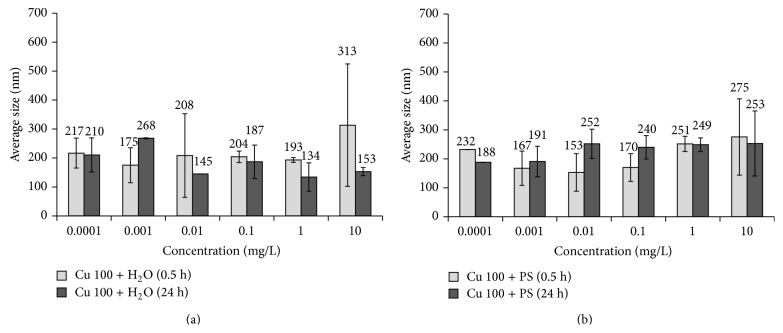
Changes in average size of Cu 100 nanoparticle suspensions based on (a) water, Cu 100 + H_2_O, and (b) physiological solution, Cu 100 + PS.

According to the data obtained from dynamic light scattering technique storage time and concentration have more effect on degree of aggregation in Cu 100 nanoparticle suspensions than they do in Cu 50 nanoparticle suspensions. In aqueous Cu 100 suspensions when concentration increases from 0.1 to 1 mg/L *d*_av_ of the aggregates becomes 1.5–2 times smaller regardless of the storage time (Figures 6(a) and 6(b)). In concentrated suspensions with 10 mg/L *d*_av_ of the aggregates rises sharply while their storage for more than 24 hours has little effect on *d*_av_ which can be explained by the particles settling (Figure 6(a)). In PS suspensions *d*_av_ of the aggregates gradually increases together with particles concentration growth. Increase in storage time for PS suspensions resulted in no significant aggregation (Figure 6(b)).

Comparison of the toxic properties of nanoparticles of various sizes shows that decrease in nanoparticle size from 100 to 50 nm leads to sharp rise of toxicity index; this effect is especially characteristic for diluted suspension with copper concentration < 0.01 mg/L. Since aggregate size has almost now influence on biological activity variation it may be concluded that particle size decrease leads to toxicity growth because of increasing number of surface atoms which have higher activity due to unsaturated bonds. Thus, for example, nanoparticles are characterized by high surface atoms to volume ratio. This fact may be of great significance for the whole spectrum of the properties displayed by a substance including its chemical, physicochemical, and biological activity [57].

At the same time, the team obtained evidence that in 24 hours activity of Cu 50 nanoparticles decreased in comparison with that of Cu 100 nanoparticles. Since the size of the particles/aggregates does not change much (Figures 4 and 6), such difference in behavior can be explained by peculiarities of chemical composition of the surface layers of the particles composing the aggregates in water suspensions. In the work quoted previously [55] it was shown by the example of similar objects of study (electroexplosive cuprum nanoparticles) that during the first hours of exposition in water the specific rate of metallic ions release from Cu 50 nanoparticles is much lower than that from Cu 100 nanoparticles. The mechanism suggested by the authors implies that low-solubility oxide-hydroxide cuprum compounds, inseparable by centrifugation, are formed on the surface with time, while the dissolution rate differs by 1-2% wt. Thus, we can suggest that in spite of formation of a hard phase new composition on the surface of particles there still remains high content of highly toxic Cu^2+^ ions in Cu 100 suspension; these ions are responsible for high toxicity of larger particles after 24 h exposure.

### 3.3. Study of Cu^2+^ Ions Solutions Toxicity

**Figure 7:**
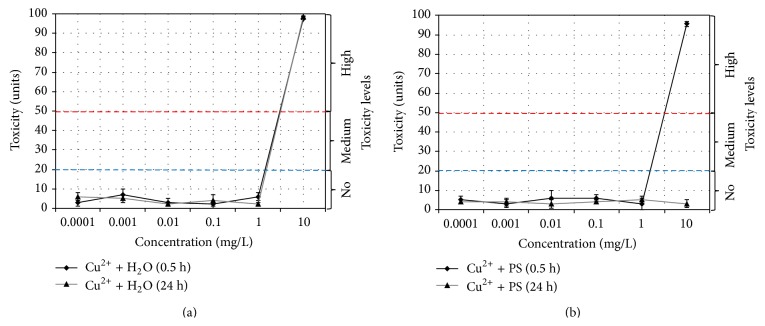
Changes in the toxicity index for Cu^2+^ solutions in (a) water, Cu^2+^ + H_2_O and (b) physiological solution, Cu^2+^ + PS.

It is deduced from experiments that Cu^2+^ solutions with concentration ≤ 1 mg/L display no cytotoxicity (Figures 7(a) and 7(b)). High toxicity level (>90 units) is observed at 10 mg/L concentration, while storage time influence upon bactericidal effect is noted only for PS-based solutions: toxic effect disappears after 24-hour storage. Minimum inhibitory concentration (MIC) for water solutions and for fresh suspension based on physical solution is 2 mg/L. Minimum bactericidal concentration (MBC) for water solutions is 10 mg/L; for fresh suspension based on physical solution it is 10 mg/L. For 24-hour suspension based on physical solution neither MIC nor MBC were calculated.

Change in pH level of the solutions has no influence on their toxicity value as the highest deviation from the norm produces no biocidal effect (Table 4).

One can reasonably suggest that on the one hand high toxicity of Cu^2+^ solutions at 10 mg/L concentration can be connected with reaching some threshold concentration of Cu^2+^ ions when high concentration gradient leads to increasing amount of ions diffusing into the cell through cytoplasmic membrane. On the other hand, aqueous Cu^2+^ solutions in neutral and weak basic media are characterized by some degree of instability. According to pH measurements pH values decreased from 7.2 to 6.3⋯6.6 units (Table 4), which had to increase the hydrolytic stability of the solutions. With increase in the solution concentration up to 10 mg/L the concentrations product Cu(OH)_2_ will exceed the dissociation constant; consequently the deposited amorphous particles of copper hydroxides can adhere to the cell surface leading to membrane permeability deterioration thus disrupting the cell life functions.

### 3.4. Control Toxicity check In Bactericidal Solution of SDC

**Figure 8:**
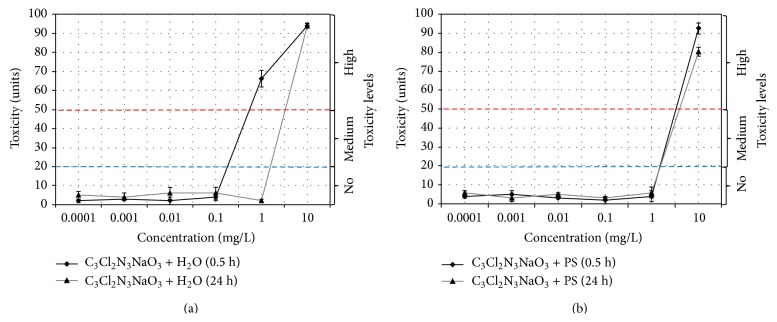
Changes in the toxicity index for SDC solutions (C_3_Cl_2_N_3_NaO_3_) in (a) water and (b) physiological solution (PS).

The control study of the influence of widely employed antibacterial agent shows that toxicity index has negative or zero values at substance concentration < 1 mg/L. It can be decisively stated that only at concentrations exceeding this value almost complete biosensor luminescence quenching is observed in all the studied media while storage time has no notable effect on the product antimicrobial properties (Figures 8(a) and 8(b)) and on initial pH value of 7.2.

Minimum inhibitory concentration (MIC) for fresh water solution is 0.25 mg/L, and for 24-hour suspension it is 2 mg/L. For suspensions based on physical solution it is 2 mg/L. Minimum bactericidal concentrations (MBC) for freshly prepared and 24-hours water solutions and for fresh suspension based on physical solution was 10 mg/L. MBC was not evaluated for 24-hour suspension based on physical solution within the studied range of concentrations.

## 4. Conclusion

Thus, the present work displays the influence of such factors as storage time, composition, and concentration of copper-containing solutions and suspensions upon their bactericidal effect against* E. coli* M-17 bacteria.

Comparative analysis of the influence of various characteristics of Cu 50 and Cu 100 nanoparticle suspensions upon their performance against* E. coli* enabled us to determine the most efficient concentrations (mg/L) displaying the highest antibacterial effect (Table 5).

It was determined that reduction of nanoparticles initial size from 100 to 50 nm results in rise in their antimicrobial activity; at the same time character of the dependence of toxicity on concentration also changes as toxicity peaks at low concentrations (0.0001⋯0.01 mg/L) are observed. The obtained results correspond with the available data on increased toxicity of nanoparticles of smaller size [58–60] and on nonlinear character of nanotoxicity, which may result from such factors as step-like character of adaptation of living organisms to stresses, signaling role of low-intensity nanoparticle influence, nonlinear stochastic resonance induced by weak influences [61, 62].

Furthermore, no influence of medium-sized nanoparticles/aggregates in suspensions upon their antibacterial properties was noted. It was shown that the suspensions storage time is an important factor altering antimicrobial properties of small nanoparticle suspensions of Cu 50 where toxicity decreases after 24-hour storage, while suspensions of larger Cu 100 nanoparticles display no such dependence.

It was found that dispersion medium has different influence on antibacterial properties of suspensions containing nanoparticles of different sizes: unlike Cu 50, high-concentration suspensions of Cu 100 nanoparticles (10 mg/L) display higher toxicity when physiological solution is used instead of water.

The conducted experiments enabled us to detect differences in toxicological effects of nanostructured and ionic forms of copper. In large, Cu^2+^ solutions display lower level of toxicity at concentrations of 0.0001⋯1 mg/L than Cu 50 nanoparticle suspensions. Although at the maximum concentration of 10 mg/L copper in ionic form shows the highest toxic effect comparable with that of antimicrobial product based on aqueous solution of C_3_Cl_2_N_3_NaO_3_.

The received results can be used for creating new types of antibacterial products based on nanoscale copper particles as well as for development of methods for toxicity forecasting and evaluation of aqueous solutions containing copper.

## Figures and Tables

**Table 1:** Values of oxidation film thickness for cuprum nanoparticles.

Sample	Oxygen content, %	Oxidation film thickness, nm
Cu 50	5.1	0.6
Cu 100	6.3	1.5

**Table 2:** рН value of Cu 50 nanoparticle suspensions.

Concentration (mg/L)	Cu 50 + H_2_O (0.5 h)	Cu 50 + H_2_O (24 h)	Cu 50 + PS (0.5 h)	Cu 50 + PS (24 h)
0.0001	7.6	7.0	7.2	7.1
0.001	7.6	7.1	7.2	7.1
0.01	7.3	7.1	7.1	7.2
0.1	7.3	7.2	7.1	7.2
1	7.3	7.2	7.2	7.2
10	7.4	7.2	7.3	7.2

**Table 3:** pH value of the studied Cu 100 nanoparticle suspensions.

Concentrations (mg/L)	Cu 100 + H_2_O (0.5 h)	Cu 100 + H_2_O (24 h)	Cu 100 + PS (0.5 h)	Cu 100 + PS (24 h)
0.0001	7.0	7.1	7.0	7.4
0.001	7.2	7.1	7.1	7.4
0.01	7.3	7.2	7.1	7.5
0.1	7.3	7.2	7.1	7.4
1	7.3	7.2	7.1	7.4
10	7.2	7.4	7.7	7.2

**Table 4:** pH levels of Cu^2+^ solutions.

Concentrations (mg/L)	Cu^2+^ + H_2_O (0.5 h)	Cu^2+^ + H_2_O (24 h)	Cu^2+^ + PS (0.5 h)	Cu^2+^ + PS (24 h)
0.0001	7.2	7.2	7.1	7.2
0.001	7.2	7.3	7.2	7.2
0.01	7.2	7.3	7.1	7.3
0.1	7.2	7.2	7.1	7.2
1	7.1	7.1	7.0	7.1
10	6.7	7.1	6.6	6.3

**Table 5:** The most efficient concentrations (mg/L) displaying the maximum antibacterial effect.

Conditions: media and storage time	Nanoparticle suspension	Nanoparticle suspension	Cu^2+^ solution	C_3_Cl_2_N_3_NaO_3_ solution
	Cu 50	Cu 100		
H_2_О 0.5 h	0.0001; 10^*∗*^	—	10	1; 10
H_2_О 24 h	—	—	10	10
PS 0.5 h	0.01; 10^*∗*^	10	10	10
PS 24 h	—	10	—	10

^*∗*^Nonlinear toxicity effects.
